# Destination Amyotrophic Lateral Sclerosis

**DOI:** 10.3389/fneur.2021.596006

**Published:** 2021-03-29

**Authors:** Matt Keon, Benjamin Musrie, Marcel Dinger, Samuel E. Brennan, Jerran Santos, Nitin K. Saksena

**Affiliations:** ^1^GenieUs Genomics Pty Ltd., Sydney, NSW, Australia; ^2^School of Biotechnology and Biomolecular Sciences, University of New South Wales, Sydney, NSW, Australia; ^3^Advanced Tissue Engineering and Stem Cell Biology Group, School of Life Sciences, University of Technology Sydney, Sydney, NSW, Australia; ^4^Epigenes Australia Pty Ltd., Melbourne, VIC, Australia

**Keywords:** neuromuscular disease, motor neurons, neurodegeneration, pathophysiology, early signs, gene-environment interaction

## Abstract

Amyotrophic Lateral Sclerosis (ALS) is a prototypical neurodegenerative disease characterized by progressive degeneration of motor neurons both in the brain and spinal cord. The constantly evolving nature of ALS represents a fundamental dimension of individual differences that underlie this disorder, yet it involves multiple levels of functional entities that alternate in different directions and finally converge functionally to define ALS disease progression. ALS may start from a single entity and gradually becomes multifactorial. However, the functional convergence of these diverse entities in eventually defining ALS progression is poorly understood. Various hypotheses have been proposed without any consensus between the for-and-against schools of thought. The present review aims to capture explanatory hierarchy both in terms of hypotheses and mechanisms to provide better insights on how they functionally connect. We can then integrate them within a common functional frame of reference for a better understanding of ALS and defining future treatments and possible therapeutic strategies. Here, we provide a philosophical understanding of how early leads are crucial to understanding the endpoints in ALS, because invariably, all early symptomatic leads are underpinned by neurodegeneration at the cellular, molecular and genomic levels. Consolidation of these ideas could be applied to other neurodegenerative diseases (NDs) and guide further critical thinking to unveil their roadmap of destination ALS.

## Introduction

In general, the start points in a given disease are defined as the points in time where the patient presents to the clinician, while the endpoints are the ones where specific outcome are reached, or the disease is cured. We seldom back-track a disease to fully understand where it commenced. In chronic diseases, we invariably miss the actual start points and the tell-tale signs because these initial signs generally do not exceed a certain threshold and, thus, are livable. Due to the less pronounced early symptoms and their limited impact on daily life, they are ignored by those affected, and when they finally present to a clinician they are often not communicated. By not starting at the first start point, a start-to-finish roadmap of a disease will always be inaccurate - in this case, the roadmap of Amyotrophic Lateral Sclerosis (ALS).

### What Is Amyotrophic Lateral Sclerosis (ALS)?

ALS is known to be one of the most common and aggressive neuromuscular diseases, which can hit hard following the subtle early signs that are often overlooked and lead to one of the most debilitating illnesses. Most commonly, it strikes people between 40–60 years, but also younger individuals can develop ALS. Two point six individuals/100,000 develop ALS every year, whereby men generally have a higher propensity to develop ALS than women (1).

ALS slowly takes away the ability to walk, eat, or breathe from those affected. This devastating adult-onset neurodegenerative disease leads to the loss of motor neurons, the long nerve cells in the brain (upper motor neurons, UMNs) and spinal cord (lower motor neurons, LMNs), which innervate the muscles (2). These motor neurons are essential for the communication between the brain and the muscles and transmit vital instructions for mobility. When these nerve cells are dysfunctional or damaged, they gradually stop communicating with the muscles, and the brain loses its ability to control and initiate voluntary movements such as walking, chewing or talking. This results in a progressive weakness, muscle twitches (fasciculations), and atrophy of voluntary skeletal muscles throughout the body. In the final stages of the disease, this leads to fatal paralysis and death due to respiratory failure.

### The Current Views and Paradigms of ALS

Chronic diseases, such as neurodegenerative diseases (NDs), including ALS, are different from infectious diseases where, if the source of infection or epidemic can be back-tracked, the resolution to the problem generally becomes clearer, and targeted treatment can be applied. There is no global consensus on the criteria that define the start point of ALS in humans or preclinical studies involving mouse models of ALS. Nevertheless, no disease can begin without a start point. However, the same disease can have heterogenous start points that vary from person to person and follow numerous roads to the same endpoint, which is the case with ALS. This can be illustrated by metro lines where the map is different for each line, but due to intersecting stations between different maps, the metro lines can still end at the same destination. Thus, identifying these intersections, which are the genes and pathophysiological pathways, is vital in elucidating the map that leads to ALS. Moreover, it is important to emphasize that there is a serious debate in relation to pathological and clinical and phenotypic heterogeneity and whether this means that ALS should be treated as a single entity, or if it needs stratification relying on molecular signatures that are only partially reliable given their complexity (3, 4).

While recent advances in the understanding of ALS have led to new questions (5), transgenic mouse models have failed to provide rapid advances in prevention and treatment of ALS and masking the true complexity of ALS disease in monogenic models. As reviewed by Turner and Swash (5), ALS has evolved into a multisystem disorder, which may involve a final common pathway and clinical core accessible via multiple upstream etiological tributaries. Continuing clinical observations, investigating the molecular complexity of ALS, and the convergence of the resulting findings are critically important to inform the development of new therapies or prevention strategies (5). This is the main premise for this review. It can be best explained by an example of C9ORF72 mutation and the primary assaults it incurs and the connections each of these assaults maintain with multiple cellular processes in impairing them eventually leading to and converging on neurodegeneration. Recent studies have attempted to understand how the repeat expansion of GGGGCC hexanucleotide disrupts cellular physiology, and have suggested convergence on downstream, functional defects in cells, such as nucleocytoplasmic transport disruption, membrane-less organelle defects, and DNA damage, and repeat RNAs and DPRs, etc. Defects in one cellular organelle or processes usually disrupt others. Indeed, many cellular functional defects in C9ALS/FTD are connected, and all these functional connections maintained among cellular deficits cause functional impairment of these processes and lead to neurodegeneration, which is at the heart of this review (6). This study by Tang et al., has not only provided an integrated view of the disease mechanism but also revealed novel cell biology implicated in neurodegeneration through convergence of insults incurred by C90RF72 mutation in ALS patients.

In our review many such hypotheses have been discussed to strongly and logically bring about the issue of convergence of various deficits leading to neurodegeneration, which is central to ALS and other neurodegenerative diseases. The main etiological factors that lie at the heart of neurodegeneration and influence degenerative processes either singly or collectively, include oxidative stress, mitochondrial damage, glutamate excitotoxicity, defective axonal transport, glial cell impairment, impaired DNA and RNA metabolism, autoimmune responses, metabolic impairment, heavy metal toxicity, viral infection, apoptosis, proteinopathies, and proteome homeostasis (2, 3, 7).

If we take this new paradigm that ALS does not start as a multifactorial or multisystem disease, but evolves into one, we can get a more informative picture and a clear roadmap of ALS. Every early tributary and its identification, before it forms tracks, is significant at early stages. Further, recent studies have proposed ALS to be a multistep disease, and these steps may be different across patients. An incidental analysis and identification of a reduced number of steps in patients with ALS with genetic mutations compared to those without mutations support the idea of ALS as a multistep disease process (8, 9).

### Unraveling ALS From Its Point of Origin

The main question is, what triggers ALS? Which modality starts first before the disease becomes multifactorial? Is there a single thread that starts the disease and leads to a cascade effect resulting in a multifactorial disease? Is the first start point or single thread always the same, or are there diverse start points leading to the same ALS endpoint? Moreover, if there are diverse start points, are their functional correlates related? Are these etiological factors related, and what functional threads do they maintain during the disease evolution?

To answer these questions, it is imperative to understand the holistic functional interaction between the various aforementioned etiological factors and the pathways they influence. For the convenience of understanding, researchers have looked at these entities singly, which has hampered the broader understanding of ALS. Various hypotheses proposed hover around single factors, failing to reconcile that these factors maintain tight and wider functional connections as the disease progresses, but are not evident at the start. We need to begin by understanding the early onset before going into the details of later aspects of disease progression. By identifying these early symptoms and considering the underlying pathophysiological mechanisms in the design of new therapies, we may hopefully prevent the disease progression and the emergence of further manifestations and cure the damage that has already occurred. Of course, this would lie at odds with the single target idea of ALS, but as chasing single targets as the genesis of ALS have not furthered the field in terms of therapeutic outcomes, it is time to open up and broaden our thinking. At present, it is difficult to identify the onset of the disease other than through observational study on ALS patients coinciding with the onset of focal weakness, wasting, or mental change. The main problem with this is that these onset features vary and are vague in their timing. However, we must take note of these early alterations as they may represent phenotypic changes that exist sub-clinically for many months or even years before the disease develops (10). Thus, the study of pre-symptomatic individuals may shed light on the early preponderance of highly-penetrant ALS gene mutations (11).

## The Disease

### What Causes ALS?

ALS is no longer viewed as one disease or a disease with a single unified cause. It is now considered a clinicopathological syndrome, caused by a complex convergence of environmental influences coupled with genetic susceptibility and age-related loss of cellular homeostasis, leading to neurodegeneration (12).

In 1824, the surgeon and philosopher Sir Charles Bell first described a rare condition, the clinical features of which matched those of ALS (13). It was not until 1874, when the neurologist Jean-Martin Charcot gave the disease its modern name and described it as a neurological problem of a mysterious nature (14). Although ALS had already been discovered in the 19th century, the causes and the basic underlying mechanisms of ALS remain obscure (15). Currently, no durable treatments exist that can prevent, reverse, or even alter the course of ALS, with recent drug candidates (Riluzole and Edaravone) (16, 17) providing extremely modest increases in life expectancy of those affected. Even if we already knew the causes of ALS, the sheer complexity and heterogeneity of the disease challenge the development of optimal treatments. ALS patients seem to be distinct from each other, similar to the variability we see in cancer patients that may have molecularly and genetically distinct forms of the disease. Thus, targeted therapies need to be developed and tailored to the affected individuals.

It would be inaccurate to say that nothing has been learned about ALS since it was first described, but unfortunately, it is true that even after 2 centuries, the start points in ALS pathology remain a mystery. The knowledge we have acquired through research has not yet yielded dividends in terms of clinical breakthroughs that can make a difference in the quality of life of ALS patients (18). This could be largely attributed to the heterogeneity in the start points that initiate disease in different individuals, implying that a combination of diverse factors (endogenous and/or environmental) participate in progressive motor neuron stress. This progressive stress culminates in the activation of aforementioned pathways that incur insults on several neurological compartments encompassing the central and peripheral nervous system. Thus, it is logical to suppose that the interactions between genetic and environmental risk factors lead to degeneration of the neuromuscular junctions (NMJ), which is a hallmark of ALS onset and pathogenesis. To explain this further, the neuromuscular junction assembly and their plasticity is tightly regulated, through a cross-talk between motor nerve endings, muscle fibers and glial cells, at all stages of life starting from embryonic, postnatal to adult life. Any alteration in their ability to communicate is possibly responsible for its destruction in pathological states. Thus, the neuromuscular junction dismantling plays an important role in the onset of ALS. Further, the insults on the neuromuscular system overtime results in musculature weakness and atrophy leading to gradual paralysis, and death from respiratory failure, which typically happens in ALS within 2–3 years from the ALS disease onset (19).

### Early Signs and Pathogenic Process in Motor Neurons and Disease Progression

#### Early Onset of ALS – What and Where to Look?

“Hit hard, hit early” applies to any disease, including ALS. However, we have a poor understanding of the early signs that are not disease-specific, generally do not exceed a certain threshold, and are often ignored. Numerous NDs that afflict humans start years or decades subliminally before any symptoms are noticed, and ALS is no exception (20). According to Eisen et al., ALS may even start at birth, because early programming of metabolic abnormalities that shape a disease can take decades before a full-blown disease phenotype is expressed or any structural change is visible (10).

ALS, being a non-cell-autonomous disease, further complicates the issue of diffuse, non-specific, easy to ignore symptoms. Over the course of the disease, the degeneration and death of motor neurons leading to skeletal muscle denervation will progress, likely aided by inflammatory signaling by glial cells and other cells of the peripheral immune system. Degenerative nerve diseases are known to affect many activities of the human body, including talking, breathing, chewing, swallowing, balance, movement, and heart function. Although the causes for these degenerations in NDs (Amyotrophic lateral sclerosis, Alzheimer's disease, Parkinson's disease, Lewy body Spinal muscular atrophy, Huntington's disease, and Friedreich's ataxia disease) are not known, it is believed that toxins, chemicals, and viruses can trigger this process, which becomes progressive over time (21).

Elucidation of interactions between cellular degeneration and system-level degeneration will provide the much-needed disease map in its earliest symptomatic phases, and based on this understanding will lead to the improved development of relevant biomarkers for disease progression and subsequently to targeted treatments, in addition to addressing the biological basis of disease heterogeneity at the clinical and molecular levels.

#### Early Symptomology and Indications

There are several common early clinical symptoms, all of which are underpinned into muscles, neuronal dysfunction or neuronal degeneration resulting in the loss of voluntary movement in almost all NDs, including ALS:

- **Loss of Coordination** is one of the first signs of ALS, starting slowly and increasing in frequency over months or years before becoming mingled with other symptoms. It is attributed to the damage to the nerve paths connecting the brain to the spinal cord (18).
- **Muscular weakness** occurs as a result of motor neuron loss and a lack of signaling to muscle fibers (18).
- **Vocal pitch changes** are often seen in ALS patients and are attributed to Laryngeal dysfunction. Laryngeal dysfunction occurs due to the loss of neurons, which affect the bulbar nerves (18).
- **Slurred speech or Dysarthria** is different from Laryngeal dysfunction and is caused by the loss of motor function. Speech is slurry, and fluctuations in the volume are uncontrollable because of lack of coordination in speech muscles, and, as a result, the movement of the muscles around the mouth may be exaggerated (18).
- **Cramps and muscle twitching** are an early warning sign of ALS that is often ignored, and it is a consequence of nerve-endings pressing against the muscles. The fasciculation, which is one of the most prominent features of ALS, is itself evidence of the increased excitability at the level of LMNs (22).
- **Uncontrollable laughing and crying** (**pseudobulbar affect**) occur when nerve degradation leads to improperly inhibited emotions (18).
- **Breathing difficulties** (such as increased breathlessness, shortness of breath, breathing discomfort) are the first and foremost reason for short survival. Death is often attributed to a loss of control of the respiratory muscles. This diaphragmatic dysfunction is caused by marked loss of motor units leading to weak inspiratory strength, respiratory fatigue, hypercapnia, and hypoxemia. Breathing problems do not occur immediately but affect most ALS patients, eventually resulting in death (23).
- **Problems swallowing (Dysphagia)** is a common symptom seen at late stages of ALS and is caused by the lack of muscular control that is guided by the nerve cells that control muscle movements. Predominantly, the ALS patients with bulbar involvement demonstrate severe swallowing difficulties, but dysphagia is also seen in non-bulbar involvement ALS patients. Bilateral degeneration of the UMNs in the primary motor areas also impairs other motor areas, leading to a substantial reduction of “swallowing related” cortical activation (24).
- **Weakness of the neck muscles** is seen in almost all ALS patients at some stage, and this inability of the muscles to support head results into “dropped head syndrome,” and at the extreme, the patient will be unable to look straight ahead. This is attributed to the deterioration of cervical paraspinal extensor muscles at the back of the neck (25).

While the subliminal signs serve as an early warning system in predicting the disease, the transition from pre-symptomatic to symptomatic phases of the neurodegenerative process implies possible intervention of pathogenic mechanisms which must be different from the starting events (26). Thus, to design a therapeutic intervention, a critical understanding of these transition states in necessary so they can be stopped in tracks rather than progressing to the next stage. This is what is critically lacking in defining effective treatments for ALS.

### Neurodegeneration Is the Core of ALS

As with other NDs, the symptomatology described above shows that neurodegeneration is the core event that causes all symptoms of ALS, in addition to many NDs as well. The neurodegenerative process in ALS is characterized by the loss of motor neurons (i.e., the nerve cells that regulate voluntary muscle movement). This loss leads to muscle weakness and muscle wasting throughout the body because of the degeneration of both the UMNs and LMNs.

In support of these symptoms, it is important to mention that the evidence emerging from autopsies demonstrating ALS being a cerebral pathology is associated with regions beyond the primary motor cortex (9). Cognitive impairment, occasional psychosis, and subtle dysexecutive neuropsychological symptoms were recognized in earlier studies, followed by more recent positron emission tomography supporting strong cerebral involvement in ALS (27–29). Furthermore, ubiquitinated inclusions of the protein transactive response DNA binding protein 43 kDa (TDP-43) are found in both ALS and frontotemporal dementia (FTD) (30). Notably, overt FTD manifests early in about 10–15% of patients with ALS (31), and seems to be strongly associated with a G4-C2-hexanucleotide repeat expansion in chromosome 9 open reading frame 72 (C9*orf* 72) (32). Together this link between ALS and FTD not only extends ALS as a motor system disease into the frontotemporal lobes but may also connect with early symptomatology related to balance, expression of thought, planning, personality, and speech, in addition to motor symptoms. The clinically distinctive presentations of ALS might be viewed as a failure of several evolutionarily-interlinked functions, namely upper limb functions, in particular hand functions, linked to the development of bipedalism (involving changes in corticomotoneuronal connections), impaired vocalization, swallowing, and breathing (involving the brainstem functional complex); and FTD involving selective impairments of cognitive functions linked to socialization (33). This leads to the suggestion of the presence of discrete systems whose separation defines the ultimate expression of the neurodegenerative process. Thus, at early stages when these symptoms appear, selective vulnerability (a long-held view) of neurons can give clues on what next to come, which has given birth to the idea of ALS being a multisystem disease primarily dictated by the properties intrinsic to the motor system and the individual neuron (12). Indeed, early comparisons of healthy and neurodegenerative functional connectome of the brains provide valuable insight into the evolution of symptoms and neurodegeneration (34).

### Key Tenets of Neurodegeneration

Neurodegeneration is a central tenet of ALS, and a striking estimation is that one-third of large motor neurons must be lost before there is any visible atrophy (35).

Both UMNs and LMNs are involved in ALS, and the progressive degeneration of the motor neurons eventually leads to death in ALS patients (5). Motor neurons are unique and vital cells. They are the longest cells in the body with high energy demands and are responsible for integrating signals from the brain and the sensory systems to control voluntary and involuntary movements.

To maintain a seamless communication between brain and body, axon structure and dynamics are vital to drive retrograde and anterograde transport of material along the length of motor neurons. The cargoes of the transport proteins influence many functional and developmental pathways in motor neurons such as the axon guidance pathway, metabolism, energetics (ATP synthesis) and mitochondria, apoptosis, excessive glutamate release, and neuroinflammation. A disruption in any of these processes is implicated in neurodegeneration. Notably, given their extreme size and energy demands, motor neurons are prone to be directly impacted by dire consequences from malfunctions in any of these pathways (36). Furthermore, the functionality and survival of motor neurons usually are intrinsically supported by different types of glial cells. Consequently, glial cells also play an integral role in neurodegenerative processes (37).

During disease progression, neuronal degeneration spreads to other brain regions, and the pattern of this degenerative process is often domino-like (38). It has been suggested that the ripple effect is based on the disruptive effects of neuronal dysfunction and death on both pre- and postsynaptic neurons (38). Neurotransmission is a highly energy-dependent process, and, thus, synapses are the most vulnerable regions of neurons. The differences among synapses in their structure, metabolism, and signaling mechanisms might act as determinants of neuronal vulnerability (39). The biochemical, structural, and genetic changes that occur in the cellular milieu in which neurons reside (including glial cells (astrocytes, oligodendrocytes, Schwann cells, microglia) and vascular cells), considerably influence the fate of neurons in NDs. Thus, it is pivotal to investigate the intrinsic and extrinsic factors that contribute to the vulnerability of neurons need and to delineate their role in disease pathology.

As neurodegeneration is similarly central to other NDs (such as Parkinson's disease, Huntington's disease, and Alzheimer's disease), this process can be best understood when visualized in parallel with other NDs. Although in each disease, specific neuron subclasses in specific brain areas are affected, neurodegeneration, and the resulting loss of neurons is a unifying feature of all NDs.

### Subliminal Signs of Neurodegeneration During the Emergence of a Disease Process

Mild and vague symptoms that repeatedly occur over time are rarely investigated further by patients who are affected by them. However, analogous to the observation that unconsciously perceived subliminal language is able to exert long-lasting effects on neuronal signals and durably affect neural architecture (5), the subtle prodromal symptoms may induce comparable long-lasting effects. Consequently, a greater understanding of what these symptoms implicate, and the pathophysiological mechanisms that underpin them may yield valuable insights into the early molecular events that trigger the onset of the disease and its progression and lead to improved diagnosis earlier in the disease course.

Structural and functional MRI studies highlighted that early neurodegeneration is likely compensated by increased activation of the remaining neurons in the same brain region and in a wider sense also by activation of additional parts of cerebral networks resulting in equal behavioral performance between affected and non-affected subjects (40). This explains, at least in part, how early symptoms go unnoticed or unreported for some time. However, the compensation is overridden at some point during the disease (40). This overriding process is crucial to understanding the disease as this holds the balance between manifestation and transitory non-manifestation of the disease, and is critical when the disease has paused due to unknown reasons while remaining subliminally active. There may be a molecular dynamic operating below the threshold that has a direct bearing on early clinical symptoms. It is conceivable that clinically-discernable and concerning symptoms only manifest when a molecular event exceeds a certain threshold. A common question is “what is cause and what is consequence?”. This question may be too simplistic to fully untangle the multifactorial nature of this disease. The consequences of a particular mutation or group of mutations or molecular events may become a driving cause in itself where the initial cause or event dies off or ceases. That is, the secondary events become a key player, almost like a relay where the baton gets handed over, and the race progresses forward. Indeed, one molecular process may begin where the other process or processes stop.

As this concept of functional compensation appears to emerge as a common compensatory mechanism in different sporadic and inherited NDs, early detection of the onset of neurodegeneration remains crucial as it may allow for early interventions preventing disease progression. Although neuropathology is considered the diagnostic gold standard, it is invasive and requires biopsies that can be obtained usually at autopsy. As early neurodegenerative signs are heterogeneous with respect to phenotype and underlying pathophysiological mechanisms, we need a new generation of effective non-invasive diagnostic methods detecting the diverse nuances of early signs of neurodegeneration. Such methods could facilitate the early diagnosis and, thus, the selection of appropriate available pharmacological interventions, as well as the development of new, more effective targeted therapies.

### Hypothetical Walk to Follow the Endpoints in Neurodegeneration

It is believed that both genetic and sporadic forms of ALS are a consequence of the failure of a neuronal mechanism triggered by defects in specific genes shaped by the environment over time (41). Thus, there is an interplay between genes, environment, and time, resulting in gradual insults on the neuronal system during the evolution of ALS. In the following section, various possible start points are discussed.

#### The “Dying Back” and the “Dying Forward” Hypothesis

For many years, scientists have remained tilted toward subscribing to the “dying back” hypothesis, which implies that the origins of neuronal degeneration lie at the NMJ. In this case, the degeneration of motor neurons starts at the nerve endings in the NMJ and progresses toward the cell bodies in the spinal cord (42). In contrast, the “dying forward” hypothesis suggests that the primary damage occurs in cortical motor neurons (e.g., through glutamate excitotoxicity or altered neuronal excitability) and subsequently extends in an anterograde fashion to corticospinal projections (43). The “dying forward” hypothesis is supported by the preclinical observation that corticospinal motor neuron (CSMN) death is preceded by pre-symptomatic alterations in the apical dendrites of CSMN, including changes in dendritic arbors, spine density, and spontaneous synaptic inputs (44). This finding is of particular importance because it provides a new paradigm in our thinking, as we have grown up with a school of thought that the UMNs were not critical for ALS pathology, whereby the cellular defects occur at a very early age, even before the loss of spinal motor neurons. However, despite increasing evidence, the critical importance of cortical components of motor neuron circuitry still remains underappreciated, and further studies are needed to understand if the early onset of ALS is initiated from the cortex (44). It may also be that both “dying back” and “dying forward” processes occur during disease progression.

#### Neuromuscular Junction (NMJ) Disassembly in ALS

To date, it is not yet known whether changes of NMJ in the aging muscle are primarily caused by changes occurring to the motor neuron or the muscle fiber. However, regardless of the exact progression mode, the degeneration of NMJ, leading to skeletal muscle denervation, is thought to play an essential role in the onset of ALS (45). Furthermore, alterations of the NMJ and skeletal muscle denervation appear to be the major determinants of clinical frailty and disease severity seen in patients with ALS patients (43).

The processes that lead to NMJ dismantling are still under investigation. However, there is a tight regulation of NMJ assembly, maintenance, and plasticity during embryonic, postnatal, and adult life; a process that occurs through continuous cross-talk between motor nerve endings, muscle fibers, and glial cells. Therefore, it can be assumed that impaired or altered communication among these vital components can trigger NMJ dismantling and, thus, skeletal muscle denervation (45).

#### Glial Centricity in ALS

While being previously recognized strictly as a neuronal disorder, it is known that ALS also involves all types of glial cells (astrocytes, oligodendrocytes, Schwann cells, microglia). There is evidence for the involvement of peri-synaptic (terminal) Schwann cells (44, 46). The cells that surround the NMJ play vital roles in modulating the strength of neurotransmission with muscle fibers, remodeling of NMJ, and post-injury repair (45). Therefore, early involvement of these cells in ALS and other neuromuscular disorders appears to be possible. Thus, a better understanding of early events taking place in the glial cell family and targeting them in ALS could yield dividends in obtaining effective treatments and improved outcomes. However, before targeting glial cells, we need to clearly understand the networks of glial cells, neurons, and muscle fibers, the temporal sequence of the events leading to neurodegenerations, and pinpoint the central modality in the network.

#### Ion Dysregulation, Excitotoxicity, and Energy Depletion in ALS

Age- and disease-related stressors can promote activation of biochemical cascades resulting in ion dysregulation, excitotoxicity, and energy depletion in synaptic terminals and neurites.

One example is the stimulation of glutamate receptors, which, under conditions of reduced energy availability or increased oxidative stress, leads to Ca2+ influx into postsynaptic regions of dendrites. This, in turn, can trigger apoptosis (47) In addition, among other processes, reactive oxygen species (ROS) can induce lipid peroxidation, causing dysfunction of ion-motive ATPases and glucose and glutamate transporters, subsequently leading to ion dysregulation, energy depletion, and excitotoxicity (39). Mitochondria are a major source of intracellular ROS and particularly vulnerable to oxidative stress. There is evidence that mitochondrial degeneration is directly or indirectly involved in the pathogenesis of ALS. Thus, mitochondrial dysfunctions seem to trigger ALS onset rather than being merely a product of cell degenerations (48).

Interestingly, any impairment in the genes relevant to energy processes has a ripple effect through several systems, as energy is key to neuronal communication and the survival of neurons. Moreover, motor neurons are particularly affected by disturbances of energy supply, as their energy demand is much higher compared to other cell types, because of their length and distal communication (49). However, the early signs indicating a disturbance in these processes are often ignored until the clinical manifestation. Nevertheless, targeting mitochondrial dysfunction is thought to be a valuable, novel treatment strategy (48).

## Running Hypotheses and Key Molecular Interactivity in ALS Development

### Mutation or Mutations: The Multistep Hypothesis of ALS and Neurodegeneration

Genetic discoveries have fueled excitement when scientists pinpointed a key genetic cause of the disease. These discoveries explained how genetic mutations play a vital role in neurodegenerative processes, that takes place inside the brain's motor neuron cells, thereby igniting a palpable sense of hope in ALS circles promising that the grim picture of ALS is about to change. However, as therapeutics are roadmap dependent, a clear understanding of the start-and endpoints remain vital to developing effective treatments for NDs.

As ALS is an adult-onset disorder, even in individuals with a congenital gene mutation that increases the risk of ALS, and many individuals with such a mutation remain healthy throughout their life, it has been suggested that ALS is a multistep disease (50). However, although the “multistep hypothesis” has been discussed previously, it has only recently been modeled, using the Armitage-Doll model (51) derived from cancer research, which suggests that a sequence of multiple distinct genetic events precede the onset of cancer. In the first study, Al-Chalabi, Calvo (50) assessed whether ALS incidence is consistent with a multistep process and estimated that, on average, six distinct steps are required for ALS to develop. The same group hypothesized that due to the large heterogeneity of ALS in terms of clinical presentation, progression, and outcome, the number of steps varies in specific subgroups of patients and that in individuals carrying a large effect mutation may have fewer remaining steps before ALS is established. Consequently, they used the Armitage-Doll model in a second study to test this hypothesis in genetically defined patient subgroups from a population-based Italian cohort (8). In this mathematical modeling study, Chiò et al. analyzed four major genes with mutations in ALS: hexanucleotide expansion repeat in Chromosome nine Open Reading Frame 72 (C9*orf* 72), transactive response DNA Binding protein 43kDa (TARDBP), fused in sarcoma (FUS), and superoxide dismutase 1 (SOD1). 1,077 genetically tested cases were included, of which 74 (6.9%) carried C9*orf* 72 mutations, 20 (1.9%) had SOD1 mutations, 15 (1.4%) had TARDBP mutations, and 3 (0.3%) carried FUS mutations. Notably, the number of steps necessary to induce ALS was reduced compared to cases without mutations and varied according to the mutated gene. Consistent with Al-Chalabi, Calvo (50), the number of steps identified in patients without mutation was six, while in patients with SOD1, C9*orf* 72, and TARDBP, the number of steps was estimated to be 2, 3, and 4, respectively. The estimate for C9*orf* 72 was confirmed in the same study by data from an Irish cohort (8). Notably, the sample size was particularly small for SOD1 and TARDBP; therefore, the results need to be confirmed in a larger cohort. Nevertheless, these results support the view of ALS being a multistep process and that different points of origin can converge to the same endpoint.

The majority of ALS cases (90–95%) are sporadic (sALS) with unknown causes, and only 5–10% of cases involve familial gene mutations [familial ALS (fALS)]. Thus, ALS seems to be a multistep disease that is not merely genetically determined, and the influence of non-genetic risk factors that influence the pathogenesis of ALS in concert with genetic factors need to be analyzed in future studies. Furthermore, Chiò, Mazzini (8) incorporated only the four more commonly mutated ALS genes; thus, the influence of other mutations is still unknown but might be relevant for a subtest of ALS patients. Besides C9*orf* 72, SOD1, TARDBP, and FUS, Ataxin-2 (ATXN2), Optineurin (OPTN), Valosin-containing protein (VCP), Profilin 1 (PFN1), Ubiquilin 2 and Ubiquilin 4 (UBQLN2 and 4) NIMA-like kinase 1 (NEK-1), Coiled-coil-helix-coiled-coil-helix domain containing 10 (CHCHD10), Senatxin (SETX), TANK-binding kinase 1 (TBK1), Kinesin heavy chain isoform 5A (KIF5A), and other genes have been linked with ALS (52).

Although mutations in the TARDDP gene reduce the number of steps to the onset of ALS only by two compared to individuals without mutations, transactive response DNA binding protein-43 (TDP-43), which is encoded by TARDDP, can be found in neuronal inclusions in more than 97% of sALS patients, suggesting a pivotal role of the protein in the pathology of ALS (52). Imbalance in the nucleocytoplasmic distribution of TDP-43, aberrant post-translational modifications, or aggregation lead to impairments of its normal function and cytotoxicity (52) and seems to contribute to motor neuron degeneration, which is the hallmark of ALS. TDP-43 is an RNA-binding protein (RBP), and there are many RBPs not even functionally analyzed that may have a role in ALS, as discussed below.

### Poly-Functionality of Proteins and Neurodegeneration in ALS

Besides the many start-points in ALS and neurodegeneration, and the multistep processes leading to disease onset, the polyfunctionality of gene and protein entities that can be perturbed by exogenous (environmental and infectious) and endogenous neurophysiological factors contributes to the complexity of ALS.

**Figure 4:**
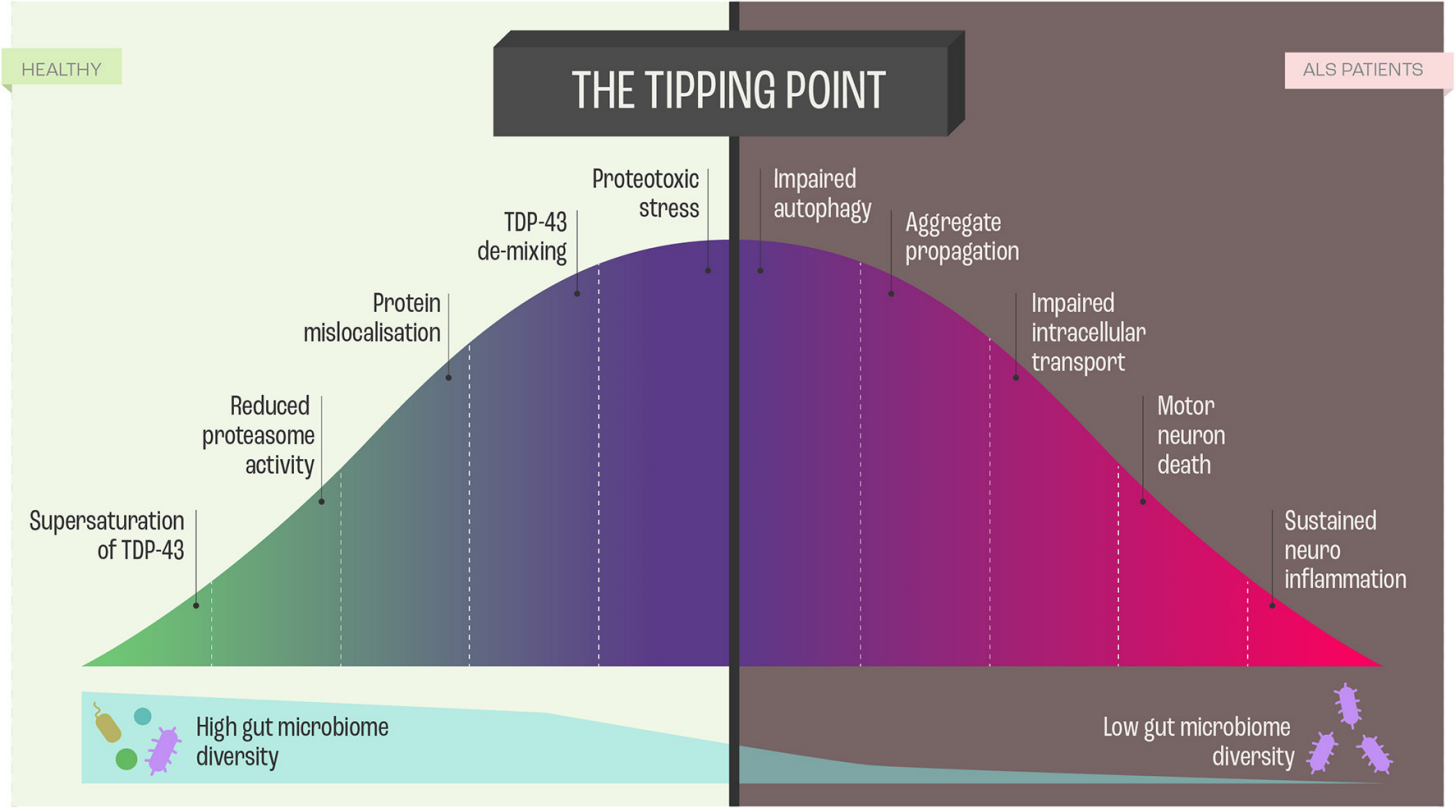
Proteome Homeostasis and functional consequences of this imbalance in Amyotrophic Lateral Sclerosis (ALS). Dysregulation of proteome in ALS is a unifying feature, and the imbalance results from processes, such as transcription, translation, mRNA metabolism, cellular trafficking, autophagy -all leading to neurodegeneration and cell death via perturbed homeostasis.

In the context of molecular etiology, TDP-43, FUS, and SOD-1, have dominated the literature on ALS pathology. These proteins are highly prone to aggregate and form intracellular inclusion bodies in the disease-affected neurons (53). The coaggregation and inclusions seem to be caused by a set of supersaturated proteins that are metastable in motor neurons. As the protein homeostasis (Figure 4) becomes progressively impaired, supersaturated proteins cannot be maintained in their soluble states (53).

Ciryam, Lambert-Smith (53) hypothesized that the various genetic modifications to the different protein homeostasis pathways and aggregation-prone proteins that give rise to different subtypes of ALS appear to converge on a more general downstream phenomenon, namely, the collapse of proteome homeostasis (Figure 4).

In this context, it needs to be emphasized that the motor neurons maintain a unique proteome, characterizing neuronal subtype, with minimal inter-subtype differences in proteome but sufficient when it comes to profound functionalities. Interestingly, in comparison to more resistant cranial motor neurons, spinal motor neurons show a reduced capacity to maintain proteome homeostasis (54) (Figure 4). Proteome homeostasis comprises various interconnected processes maintaining the functional proteome, including adaptive mechanisms in the cell that enhance the expression of chaperones, foldases, and protein degradation mechanisms under proteotoxic stress (55). There is evidence that, in particular, perturbations in the efficacy of the protein-folding machinery contribute to the selective vulnerability of motor neurons in ALS (55). However, there are still answered questions regarding the involvement of protein misfolding, protein aggregation, and inclusion in the etiology of ALS that need to be addressed: What is the nature and function of these proteins? How do the proteins affect neuronal subpopulations? What is the time course between the decline of protein homeostasis and motor neuron functioning? Which factors trigger the perturbation of protein homeostasis? Do the different proteins guide similar pathways that lead to ALS manifestation?

**Figure 1:**
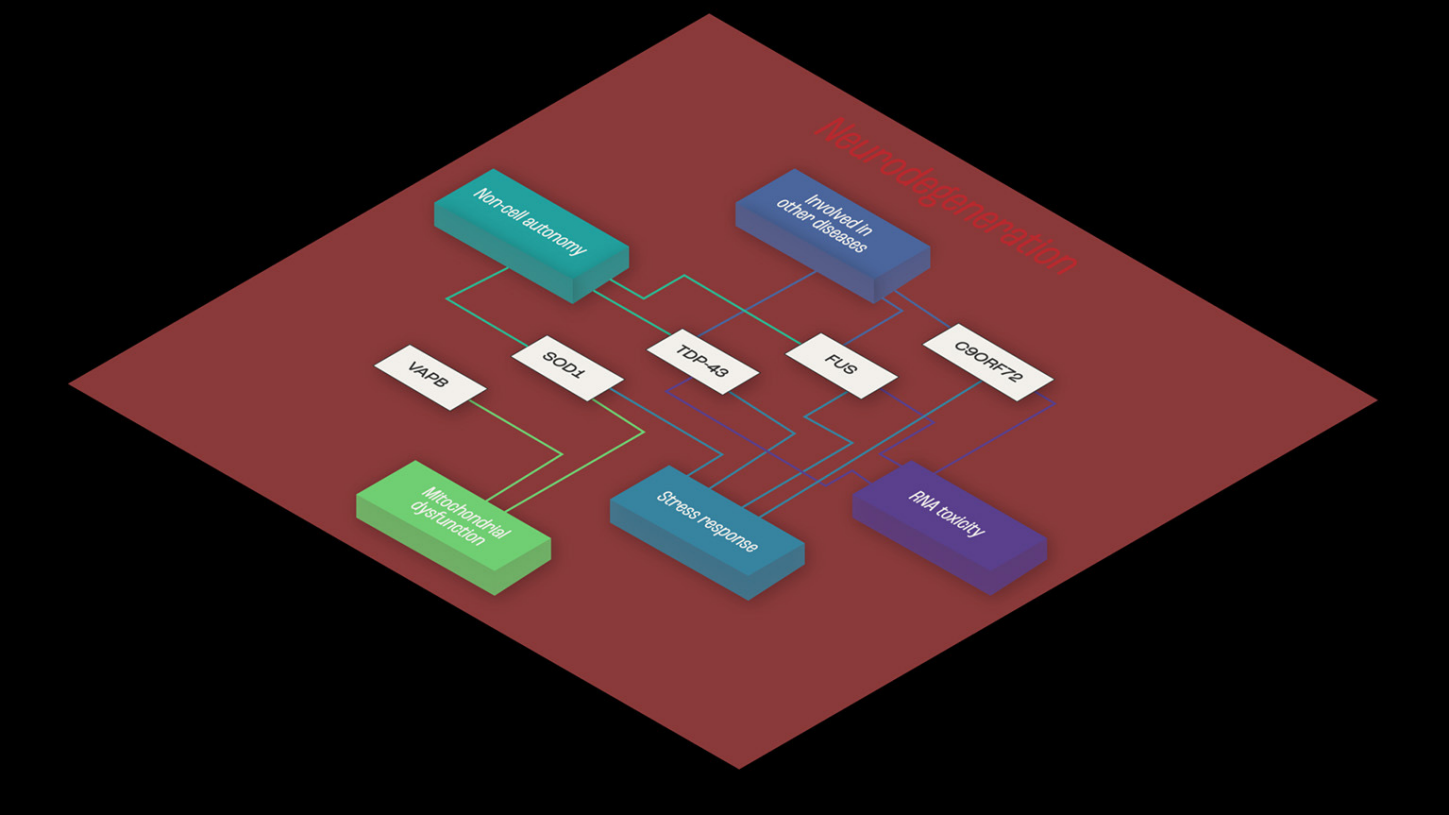
Example of functional interaction, and co-dependence. Common shared pathways in ALS, and functional similarities shared between TDP43, SOD-1, and FUS- RNA- and non-RNA-binding proteins. The three proteins appear to share mechanisms related to toxicity mechanisms and RNA toxicity, cellular stress response and mitochondrial impairment and cell autonomy, implying functional synergies in ALS disease pathogenesis.

Consistent with the multistep hypothesis, ALS is not caused by single mutations. However, it may be that co-dependent genes sharing mechanisms related to toxicity mechanisms and RNA toxicity, cellular stress response, mitochondrial impairment, and cell autonomy contribute to the onset of ALS (Figure 1).

Notably, proteome integrity and homeostasis, on which all cellular functions critically depend, are integral to maintaining synaptic plasticity and general synaptic function. Any inhibition of protein synthesis/degradation and axon transport in neurons is sufficient to trigger not only synaptic dysfunction but generalized neurodegeneration (55). The collapse of proteome homeostasis can create a ripple effect on other processes in response to both neuro-physiological and environmental triggers. Consequently, such proteins must be polyfunctional as they associate with several processes, eventually leading to neurodegeneration. They must be either highly networked or have functional synergies with other proteins that guide their successful convergence on the ALS phenotype. How this polyfunctional family of proteins influences gene networks, and how these gene networks evolve alongside environmental factors and functionally converge to cause ALS remains open to investigation.

### RNA-Binding Proteins (RBPs) in ALS

Neuronal cells have their own systems that regulate RNA metabolism (processing, localization, and expression) in response to diverse stimuli via RNA-binding proteins (RBPs), a feature that is unique to neuronal cells (56, 57).

**Figure 2:**
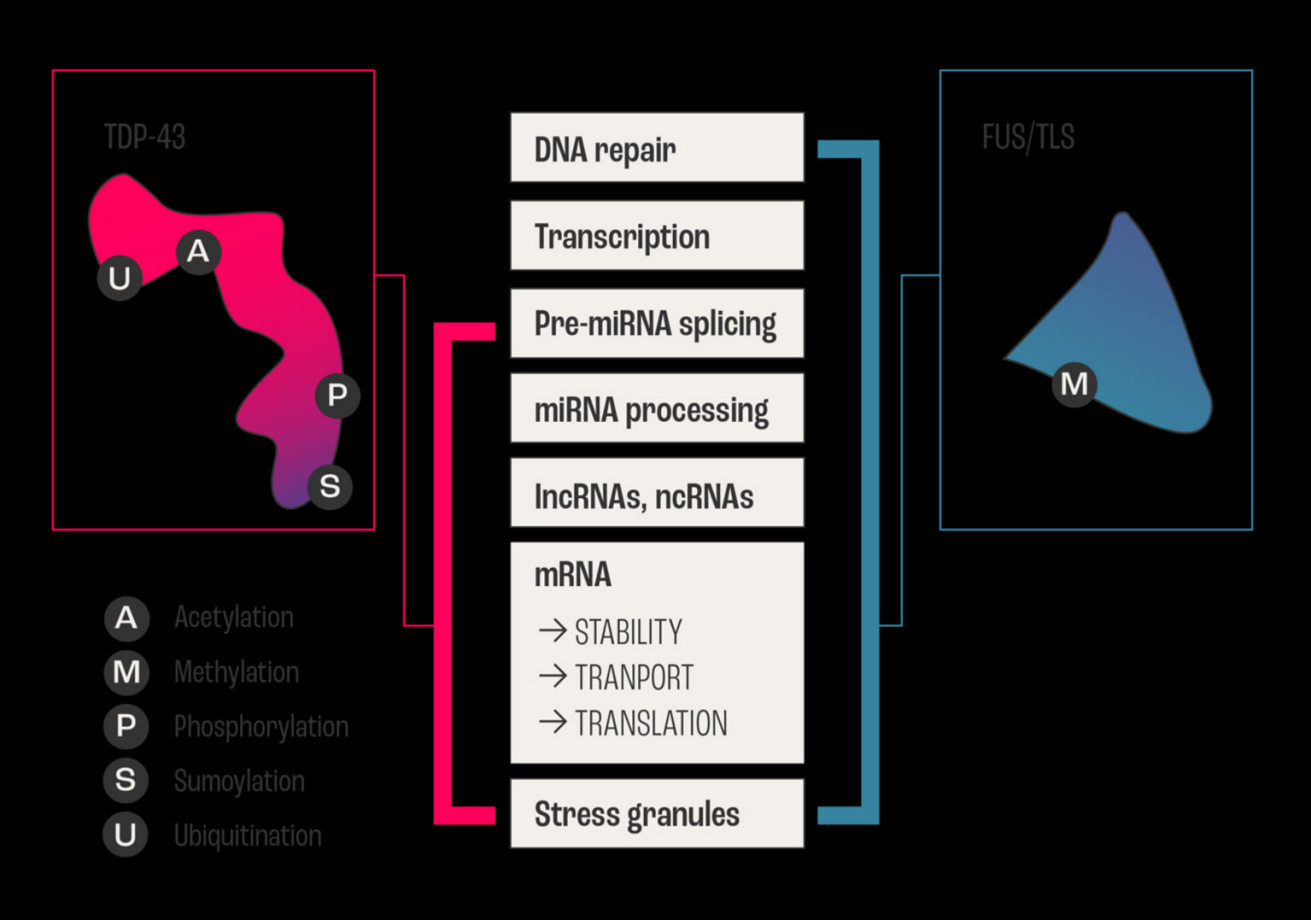
Example of overlapping and intersecting functional duties of two major RNABPs. The two major RNA Binding Proteins FUS/TLS and TDP-43 are involved in ALS, and they share regulating all aspects of RNA (metabolism, biogenesis, and life cycle) from transcription, processing, and transport/stability to the formation of cytoplasmic and nuclear stress granules. In addition to vital biochemical processes such as acetylation, sumoylation, ubiquitination, methylation, and phosphorylation within neurons. The schematic diagram shows the sharing and distribution of molecular functional between these two major proteins.

The highly polyfunctional class of proteins maintains intrinsic functional relationships between RNA and proteins (Figures 1, 2) by controlling RNA metabolism and, thus are fundamental for proteome homeostasis (57). The RBPs with roles in ALS discovered to date include TDP-43, FUS, ATXN2, TATA-box binding protein associated factor 15 (TAF15), Ewing's sarcoma breakpoint region 1 (EWSR1), matrin 3 (MATR3), T-cell-restricted intracellular antigen-1 (TIA1), heterogeneous nuclear ribonucleoprotein A1 (hnRNPA1), and heterogeneous nuclear ribonucleoprotein (hnRNPA2/B1). The RBPs share structural and functional similarities and seems that in particular mutations in low-complexity (LC) domains initiate loss of function and gain of toxic function, including increased aggregation or fibrilization propensity, cytoplasmic mislocalization, stress granule dynamics dysfunction, and finally dysregulation of RNA metabolism (58). As RBPs have common or overlapping molecular functions, molecular interactions between different RBPs, along with other non-RNA-binding proteins, are expected (Figure 1). However, mutations may alter these protein-protein interactions of RBPs in ribonucleoprotein granules, which may result in the sequestration of mRNA and subsequent inhibition of translation (54).

Furthermore, ALS-linked mutations in RBPs seem to have considerable influence on gene expression, thereby impacting cellular processes such as DNA repair response, cell growth and proliferation, and apoptosis (58). This polyfunctionality of RBPs makes it difficult to point at a single factor, pathway, mechanism, gene, or protein through which the RBPs converge to cause ALS. A clear understanding of their intrinsic polyfunctional nature, which gives them a significant advantage in creating a network with other genes and processes in guiding their unidirectional convergence, is necessary.

In this context, it is important to mention that Yerbury et al. (54) proposed a unifying view of ALS pathogenesis underpinned in the proteome. This is a significant piece of work and is tantalizing and plausible, but it does not address the elusive question of the start points. According to Yerbury and colleagues, protein production, trafficking, and degradation can all shape the proteome, and proteome homeostasis is regulated by factors influencing these aspects. Moreover, these aspects play a vital role in synaptic plasticity and general synaptic functions.

Yerbury et al.'s (54) hypothesis that “all genetic lesions associated with ALS (including in mRNA-binding proteins) cause widespread imbalance to an already metastable proteome” is consistent with the dominance of mutations in RBPs in this phenotypically diverse disease. However, it is of fundamental importance to understand how these lesions impact the genome and proteome. Moreover, the proteome of motor neurons is particularly susceptible to stress generated by homeostatic perturbations, so that early deficits in ALS may present there. The differences between motor and other neurons are relatively small, but the proteome of the motor neurons is unique. Thus, even small changes to the proteome in motor neurons may have profound functional effects, including mitochondrial dysfunction, endoplasmic reticulum (ER) stress, synapse dysfunction and altered excitability of the motor neurons, that significantly contribute to disease pathology and finally result in motor neuron death (54).

Furthermore, proteome and RNA homeostasis imbalances can affect immune pathways by becoming inert to the immune system. The way these proteins can work in an autoimmune manner points to their ability to influence various networks that contribute to the pathogenesis of ALS (59). Various modalities (Infection-Viral, Bacterial, Fungal, Bioconcentration, Autoimmune, Aggregates, Mutations) guide these processes in a dynamically complex manner.

### Gene-Environment Interaction and Neurodegeneration: The Case of the Oxidative Stress

In this context, the “gene/environmental/age-and time-dependent interactions hypothesis” for ALS is of particular importance, as it states that the risk factors operating upstream to a putative biochemical transformation which are possibly the acquired nucleic acid or protein changes that are able to result in the appearance of altered proteins or nucleic acids or abnormal accumulation of normal proteins and nucleic acids. These abnormal components can then spread within the motor system, causing downstream dismantling of the motor system with parallel biochemical, histologic, and clinical changes (62).

During this degenerative process, oxidative stress is predominant. It is known that oxidative stress and neurodegeneration are intrinsically linked because of the energy demands of motor neurons. If this process is impaired or slowed down, like in aging, the accumulation of cargo proteins over time can result in toxic effects that are reflected in both oxidative stress and neurodegeneration. As most motor neurons project downward and axonal transport is already suggested to be impaired in ALS patients, accumulated waste gradually gets deposited in axon terminals, possibly by gravity resulting in neuronal degeneration and death. This gravitational accumulation of toxic waste imposes critical energy demands, and less ATP reaches these parts, and over time causes permanent damage to motor neurons (63).

Studies evaluating a potential factor individually, along with biomarkers measuring a summation of oxidative factors, could help define their contribution to ALS risk (64, 65). Any factor favoring a pro-oxidative state could contribute to oxidative stress, and there are many indicators that oxidative stress is one of the central pathways in motor neuron disease (66, 67).

Genome and proteome interactions go hand-in-hand, and even though proteome perturbations are visible in ALS, they cannot be caused without changes in the genome. Importantly, not all genes are translated into proteins. So, while visualizing gene-protein interactions, the highly complex transcriptional-regulation of genes can be biologically significant. Deregulation in oxidative stress is the most important aspect of neurodegeneration. It could potentially be the most dominant one, as environmental risk factors [including lead (and other heavy metals), pesticides, trauma, physical activity, chronic exposure to environmental toxins, industrial chemicals, cleaning solvents/degreasers, aromatic solvents, organic pollutants, tobacco use, smoking, military service] and neurophysiological proteomic and RNA metabolism imbalances (68), contribute to changes in oxidative stress that could eventually result in neurodegeneration (69).

Further, sALS and Guamanian ALS/parkinsonism/ dementia complex (ALS/PDC) have been shown to be associated with cyanobacteria exposure, which produce neurotoxins such as β-N-methylamino-L-alanine (BMAA) (70). BMAA indirectly causes oxidative stress by inhibiting antioxidant enzymes required to combat reactive oxygen species that damage cells (71).

Consistent with the dying-back hypothesis of motor neuron degeneration, the decline in synaptic function in ALS is initiated from the presynaptic terminals. Increased oxidative stress contributes to ALS pathology by affecting the presynaptic transmitter releasing machinery, and in both sALS and fALS oxidative stress (free radical damage) is a unifying feature (72). Aberrant glutamate receptor function in astrocytes has been shown to lead to dysregulated glutamate homeostasis resulting in excitotoxicity and oxidative stress (73). Further, the aberrant activity of mutant SOD1 and TDP-43 is involved in causing oxidative damage and stress (74) in a motoneuron cell line (75). This oxidative stress can exacerbate tissue damage by interacting with other pathological events involved in promoting motoneuron degeneration. The reflection of this stress is also visible on the immune responses that are also activated in peripheral tissues of ALS patients (76). Along with environmental factors, long non-coding RNAs (lncRNAs) and RBPs are also involved in DNA damage, oxidative stress, and aging/age-related diseases, which could be due to environment-gene interactions (77).

### Genes Identified in ALS Related to Oxidative Stress and Its Associated Functional Modalities

**Figure 3:**
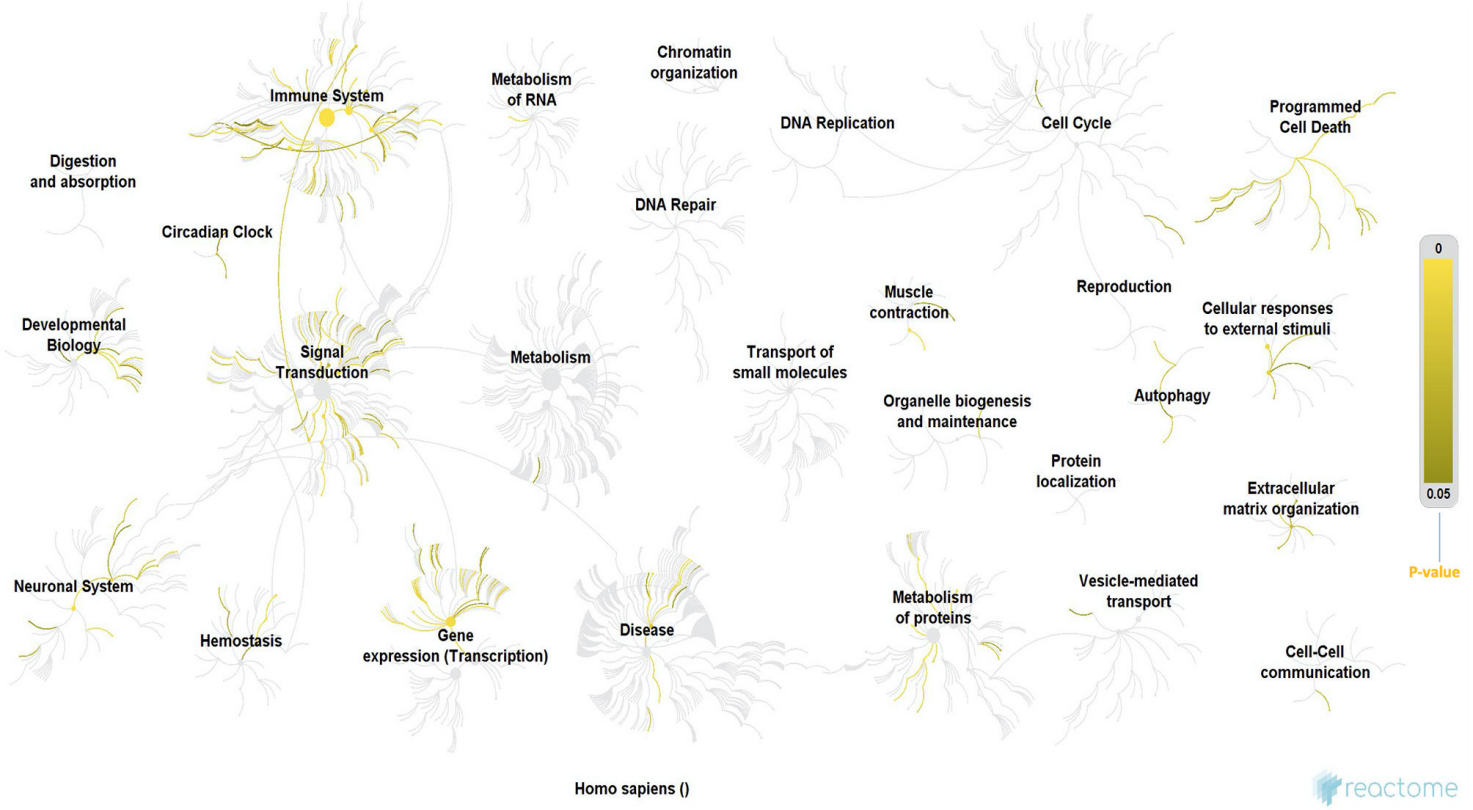
Reactome analysis of all the genes known to date in literature for their involvement in ALS. Even though these genes are not forming tigther networks between them, they still maintain cross-functionality through direct or indirect co-dependence, which also suggests that there are still unknown genes in the puzzle that may form the missing link. The processes highlighted in this Reactome analysis appear to be involved in ALS from early to late stages (60) and they form cross-functional networks that guide or impinge on the process of neurodegeneration. The color-intensity scale for *p*-values for each of the significant processes in the Reactome is shown on the right-hand side of the figure. Immune system, circadian clock, signal transduction, RNA and protein metabolism, gene expression/transcription, neuronal system, homeostasis, programmed cell death (apoptosis), muscle contraction and authophagy were some of the most significant networks in the reactome, which is consistent with various hypotheses proposed for ALS in this review, in addition to their role converging to cause neurodegeneration. **Methodology**: There were a total of 3713 mRNA genes that we looked at in the analysis. We filtered out all genes with log 2-fold changes <1 but > −1 and then focused on the up-regulated genes of which there were 576. The gene symbols of these genes were imported into Reactome, which was able to match 385 gene symbols to known identifiers. These 385 up-regulated genes were used by Reactome to perform over representation analysis which was the basis of our functional analysis. We performed this analysis on Reactome version 68 (released on 16/05/2019) (60, 61). **PS: (*****All ALS-related*** ***genes included in the Reactome analysis are listed in the*** ***Supplementary File 1*****)**.

Since the discovery of one of the major mutations in the SOD1 gene, a cause of fALS, genetic research in NDs has exponentially advanced with several hundred mutations, each defined for ALS, and other NDs. The results of our Reactome analysis of all ALS related genes (Figure 3 and Supplementary File 1), confirm the observation that the affected genes can be allocated to common themes that underlie ALS, including protein metabolism, RNA metabolism, autophagy, axonal transport, muscle contraction, apoptosis, mitochondrial dysfunction, oxidative stress, neuronal and immune system. Our analysis indicates that these mutations do not form tight functional networks but possibly maintain a functional dependence. Even though many of these pathways are not motor-neuron-specific, it is likely that the genes represented in major pathways involved in oxidation, ROS, neurodegeneration, and mitochondrial function influence motor neurons functionally through co-dependence or other unknown indirect interactions.

Furthermore, gene pleiotropy plays a significant role in ALS and other NDs, whereby one mutation in a pleiotropic gene is causing different phenotypes. For instance, the same mutations of the valosin-containing protein (VCP) gene and C9*orf* 72 appear to be present in individuals with ALS, FTD, and other neurological syndromes (78), and mutations in kinesin family member 5A (KIF5A) are associated with ALS and the Charcot-Marie-Tooth disease (79).

In addition, identical clinical indications can be caused by mutations in different genes, and polygenicity or the presence of multiple variants working in unison may cause or drive the disease in a univariate way. This is also shown by our Reactome analysis (Figure 3). While it is easy to hypothesize that these co-existent genetic variants work in concert with environmental factors, it remains unknown how these gene-environment interactions are guided temporally in shaping and directing the ALS phenotype. To verify this hypothesis, the genetic and epigenetic makeup of populations of individuals with ALS who have been exposed to specific environmental factors need to be compared to populations without such exposures. However, subjects that have been considered non-exposed ALS patients may have been exposed to one or more other factors that are able to alter similar genetic pathways, thereby guiding the ALS phenotype. This is in line with Bradley et al. (80) who hypothesized that “there are many combinations of individual, specific, genetic, and environmental factors, and that each combination can lead to the development of the syndrome of ALS” through complex gene-environment interactions (80–82).

### Gut-Brain Axis in ALS—A Hypothesis That Has Intrinsic Connection to Neurodegeneration

The human microbiome has many covert implications in human health and disease. There has been extensive interest and research on the association between the microbiome and the progression of multifactorial disorders such as NDs. ALS is such a complex disease, and a more in-depth look into the microbiome may provide some insight into its role as potential a precursor and/or progressor in ALS.

The gut-brain axis (GBA) is a bidirectional link between the enteric nervous system (ENS) and the central nervous system (CNS). There is a cascade of biochemical signaling that occurs between these two systems, and a disturbance in this signaling may have detrimental consequences on neurological health (83). The GBA signaling may be damaged by negative alterations in gut microbiota. This intestinal dysbiosis may increase the permeability of GBA and immune activation, leading to inflammation, which then may impair the blood-brain barrier (84) and promote neuroinflammation and ultimately lead to neurodegeneration (85). Additionally, internal dysbiosis also results in accumulations of misfolded amyloid and tau proteins in the brain and/or the walls of cerebral vesicles – a mechanism that is known to produce neurodegenerative effects.

A high diversity of gut bacteria is considered healthy and may be beneficial in preventing neurodegeneration. Changes in the ratio of Firmicutes and Bacteroidetes (F/B ratio) has been proposed to correlate with particular nervous system disorders (86), and a low F/B ratio in ALS patients. Additionally, short-chain fatty acids (SCFA) produced by gut bacteria play an important role in colon health and have beneficial effects on the host through their anti-inflammatory properties. An example is the SCFA propionic acid, which increases regulatory T-cells that act to suppress an immune response and positively affect the GBA and CNS by increasing remyelination. These SCFA producing bacteria are often reduced in a state of dysbiosis, which leads to an inflammatory environment (87).

The metabolites produced by gut bacteria may be one link between the microbiome and ALS. Researchers have long been using various ALS mice models to study the etiology of the disease. *Akkermansia muciniphila* (AM) is a gut bacteria strain that has received much attention, as the implementation of this strain in ALS mice significantly reduced the progression of the disease and prolonged their survival. In a study by Blacher, Bashiardes (88), transgenic SOD1 mice received AM after a 2-week antibiotic treatment. In these mice, accumulations of nicotinamide (NAM) secreted by AM were found in the CNS. The accumulation of NAM reduced ALS symptom severity and improved motor neuron functioning and altered gene expression patterns in the spinal cord of the transgenic SOD1. The subsequent analysis of the microbiome and metabolite configuration of 37 ALS human patients revealed impaired microbiome-derived NAM metabolism as well as significantly reduced NAM levels in blood and cerebrospinal fluid samples. This demonstrates the substantial effects metabolites may have on the nervous system and neurodegeneration.

The use of antibiotics has been demonstrated to eliminate gut microbes by direct or indirect mechanisms. Whilst antibiotics can be useful in eliminating pathogenic bacteria, they may also deplete beneficial bacteria and their associated metabolites that are essential for the gut, brain, and nervous system health (83). Patients with ALS may also be affected by other illnesses that require antibiotics. Finding alternative treatments to these illnesses may prove vital, as antibiotics that deplete beneficial gut bacteria may induce neuroinflammation and exaggerate ALS symptoms. Thus, it is recommended that the administration of antibiotics should be regulated in patients with ALS to assess their effect on gut flora and their subsequent effects on ALS progression.

Further research into the various strains of microbes that make up our microbiome and their metabolites will provide valuable insight into their contribution to ALS progression. Future prospective studies are needed to validate the suggested microbiome association and assess their effects on ALS disease progression.

## Discussion

This review highlights the relevance of the subtle early symptoms that are generally ignored, as they have only a limited impact on daily life. However, these less pronounced symptoms are already early signs of neurodegeneration and oxidative stress caused by various environmental, neurophysiological, and infectious stimuli. At present, it is difficult to identify the onset of ALS other than through clinical observation of the onset of early focal symptoms, including weakness, wasting, mental and physical change. Notably, these early symptoms are not only vague in their timing but are believed to appear sub-clinically many months or even years before the final diagnosis of ALS. Thus, novel effective diagnostic methods detecting the diverse nuances of early signs of neurodegeneration are needed. One possibility may be the implementation of genetic tests examining highly penetrant ALS gene mutations in individuals in a prodromal disease stage presenting with unspecific early symptoms.

To date, most studies and hypotheses consider factors and pathways apart from neuronal vulnerability to guide the neurodegenerative processes at the early and late stages of ALS, including apoptosis, excitotoxicity, calcium dysregulation, mitochondrial perturbations, and protein aggregation. Consistent with the multistep hypothesis, it seems essential to trace these heterogenous early dysregulations, which eventually converge and define the ALS phenotype. Here, the gene-environment interactions, gene-protein interactions, gene pleiotropy, protein-protein interactions are of particular relevance, as these genes and proteins identified to date in ALS do not necessarily form functional networks as shown by our Reactome analysis. Notably, these genes and proteins are able to functionally interact through cascade or ripple effects that dismantle the primary process. One example is increased oxidative stress, causing a ripple effect on normal cellular functions such as metabolism, cell respiration, gluco-and-glyconeogenesis, energy supply, mitochondrial functionality, and eventually leads to neurodegeneration – the central tenet in ALS. Consequently, deregulation in oxidative stress is largely responsible for the fate of the neurons in ALS, and that is why we see strong evidence of convergence of genes and proteins represented in these pathways. Recently, the gut-brain axis had been linked to neurodegeneration. Although first studies indicate that in particular metabolites of gut microbes may be modulators of ALS, additional studies are needed to investigate the pathological relevance.

One characteristic of ALS is the progressive loss of a selected neuronal population – the motor neurons. To date, little is known about the mechanisms leading to the selectivity of neuronal cell death, although it has been suggested that this is related to the high energy demand of the large motor neuron cells. However, future studies need to investigate why mutations in the various genes exert detrimental effects only on certain types of neuronal cells, while other cells are not affected.

## Data Availability Statement

The original contributions presented in the study are included in the article/Supplementary Material, further inquiries can be directed to the corresponding author/s.

## Author Contributions

MK conceived the idea. NS and MK wrote the article. BM provided leads on Microbiome section. MD, SB, and JS contributed to solidifying some concepts. All authors participated in reading and correcting the review.

## Conflict of Interest

MK, BM, and SB are employed by GenieUs Genomics Pty Ltd. NS is employed by Epigenes Australia Pty Ltd. The remaining authors declare that the research was conducted in the absence of any commercial or financial relationships that could be construed as a potential conflict of interest. The authors declare that the entire funding for this review was provided by the GeneiUs, Darlinghurst NSW, Sydney, Australia.
